# Myelin water imaging depends on white matter fiber orientation in the human brain

**DOI:** 10.1002/mrm.28543

**Published:** 2020-10-05

**Authors:** Christoph Birkl, Jonathan Doucette, Michael Fan, Enedino Hernández‐Torres, Alexander Rauscher

**Affiliations:** ^1^ UBC MRI Research Center University of British Columbia Vancouver Canada; ^2^ Department of Neuroradiology Medical University of Innsbruck Innsbruck Austria; ^3^ Department of Neurology Medical University of Graz Graz Austria; ^4^ Department of Physics & Astronomy University of British Columbia Vancouver Canada; ^5^ Texas Oncology Dallas Texas USA; ^6^ Department of Medicine (Division of Neurology) University of British Columbia Vancouver Canada; ^7^ Department of Pediatrics (Division of Neurology) University of British Columbia Vancouver Canada

**Keywords:** fiber orientation, myelin water imaging, quantitative MRI, T_2_ anisotropy, white matter, brain

## Abstract

**Purpose:**

The multi‐exponential T_2_ decay of the MRI signal from cerebral white matter can be separated into short T_2_ components related to myelin water and long T_2_ components related to intracellular and extracellular water. In this study, we investigated to what degree the apparent myelin water fraction (MWF) depends on the angle between white matter fibers and the main magnetic field.

**Methods:**

Maps of the apparent MWF were acquired using multi‐echo Carr‐Purcell‐Meiboom‐Gill and gradient‐echo spin‐echo sequences. The Carr‐Purcell‐Meiboom‐Gill sequence was acquired with a TR of 1073 ms, 1500 ms, and 2000 ms. The fiber orientation was mapped with DTI. By angle‐wise pooling the voxels across the brain’s white matter, orientation‐dependent apparent MWF curves were generated.

**Results:**

We found that the apparent MWF varied between 25% and 35% across different fiber orientations. Furthermore, the selection of the TR influences the apparent MWF.

**Conclusion:**

White matter fiber orientation induces a strong systematic bias on the estimation of the apparent MWF. This finding has implications for future research and the interpretation of MWI results in previously published studies.

## 1 INTRODUCTION

Myelin is a lipid bilayer membrane wrapped around the axon, protecting it from mechanical and chemical damage^1^, ^2^ and facilitating fast saltatory signal conduction.^3^, ^4^ The space between the myelin bilayers is filled with water, which is commonly referred to as myelin water. Due to reduced mobility within this confined space, myelin water has a magnetic resonance T_2_ relaxation time of approximately 10 ms at a field strength of 3 T. In contrast, the T_2_ relaxation time of the intracellular and extracellular water is in the range of 30 ms to 60 ms, and the CSF has a T_2_ relaxation time of more than 1 second.^5^, ^6^ The resulting multi‐exponential T_2_ signal decay of a white matter voxel can be measured using a multi‐echo spin‐echo scan and be decomposed into its individual components, resulting in a T_2_ spectrum.^7^ The area of the T_2_ spectrum spanning the short T_2_ components, referred to as the myelin water peak, divided by the area of the total T_2_ spectrum, is defined as the myelin water fraction (MWF). A strong correlation between the MWF and independent measures of myelin has been demonstrated in various postmortem studies.^8^, ^9^, ^10^ Over the past two decades, myelin water imaging (MWI) has been applied to mild traumatic brain injury,^11^ aging,^12^ spinal cord injury,^13^ neonates,^14^ and multiple sclerosis,^15^, ^16^, ^17^, ^18^ among others.

The human brain’s white matter is highly anisotropic. This circumstance is widely exploited in DTI, which allows us to map the brain’s complex fiber architecture.^19^ The orientation of white matter fibers with respect to the main magnetic field B_0_ also affects the magnitude and phase of the complex MRI signal and a wide range of quantitative MRI parameters. Strong orientation effects have been reported for $R_{2}^{*}$ relaxation,^20^, ^21^, ^22^, ^23^, ^24^, ^25^ gradient‐echo phase,^21^, ^24^, ^26^ and QSM.^27^ A weak orientation effect also exists for T_1_ relaxation.^28^, ^29^, ^30^, ^31^ Moreover, due to anisotropy of the cerebral vascular architecture, dynamic susceptibility‐contrast perfusion measurements conducted with both gradient‐echo^32^ and spin‐echo sequences^33^ show an orientation dependent behavior. The orientation dependency of the gradient‐echo signal of white matter is extensively studied and is ascribed primarily to the anisotropy of the magnetic susceptibility caused by the myelinated nerve fibers.^24^, ^25^, ^34^, ^35^ The observation that the T_2_ signal of white matter shows an anisotropic behavior^25^, ^33^, ^36^, ^37^ that depends on the fiber orientation, suggests that MWI is potentially affected as well. Therefore, we investigated whether and to what degree the MWF depends on the angle between the white matter fiber tracts and the main magnetic field B_0_. In the present study, we acquired MWI using a gradient and spin‐echo (GRASE) sequence,^38^ which is used widely in literature, and the Carr‐Purcell‐Meiboom‐Gill (CPMG) sequence, which is the gold standard. The CPMG sequence was further acquired at various TRs. We show that the apparent MWF depends strongly on white matter fiber orientation, independent of MRI sequence.

## 2 METHODS

Eight healthy volunteers (3 female, 5 male) with a mean age of 26 years (age range = 21‐33 years) and without any history of neurological disorder participated in this study, which was approved by the University of British Columbia Clinical Research Ethics Board. All volunteers gave written, informed consent. The MRI was performed on a 3T MR system (Ingenia Elition; Philips Medical Systems, Best, the Netherlands) using a 32‐channel SENSE head coil.

### 2.1 Magnetic resonance imaging acquisition

A 3D T_1_‐weighted sequence with TE = 4.9 ms, TR = 9.8 ms, flip angle = 8°, resolution = 0.8 × 0.8 × 0.8 mm^3^, FOV = 256 × 256 × 180 mm, compressed SENSE factor = 3.6, and acquisition time = 5:50 minutes was acquired for anatomical overview. Compressed SENSE is a combination of compressed sensing^39^, ^40^ and SENSE.^41^ For the calculation of fiber orientation, a DTI sequence with TE = 60 ms, TR = 4111 ms, b‐value = 700 s/mm^2^, 60 diffusion directions, resolution = 2.3 × 2.3 × 2.4 mm^3^, FOV = 224 × 168 × 224 mm, multiband factor = 2,^42^ SENSE factor = 2.1, and acquisition time = 4:19 minutes was acquired. The MWI was performed using the following sequences: (1) a GRASE sequence with TR = 1073 ms, SENSE = 2.5, three gradient echoes per spin echo, receiver bandwidth = 100 kHz (non‐EPI) and 350 kHz (EPI), and acquisition time = 11:29 minutes; (2) a CPMG sequence with TR = 1073 ms and acquisition time of 09:43 minutes; (3) a CPMG sequence with TR = 1500 ms and acquisition time = 13:33 minutes; and (4) a CPMG sequence with TR = 2000 ms and acquisition time = 18:04 minutes. All CPMG sequences were accelerated with a CS factor of 7,^43^ and all MWI sequences had 48 echoes with the first echo at TE = 8 ms, ∆TE = 8 ms, spatial resolution = 0.96 × 0.96 × 2.5 mm^3^, and FOV = 230 × 120 × 180 mm. The DTI and MWI sequences were acquired in axial orientation without angulation. Due to the long data‐acquisition times, not all sequences were acquired in all volunteers. A summary of all acquired sequences for each volunteer, including their acquisition times, is provided in Supporting Information Table S1. All MRI data are available from the authors upon reasonable request.

### 2.2 Image analysis

The DTI data were analyzed with the FMRIB Software Library (FSL v5.0.9).^44^, ^45^, ^46^ Distortions induced by eddy currents and head motion were corrected by FSL’s eddy_correct. FSL DTIFIT was used to calculate the diffusion tensor model, and therefore the eigenvalues and eigenvectors.

For MWI analysis, the T_2_ distributions were computed using a regularized nonnegative least‐squares algorithm with stimulated‐echo correction and a T_2_ range of 8 ms to 2.0 seconds.^6^, ^7^, ^38^, ^47^ The multi‐exponential signal decay is expressed as a T_2_ distribution, in which the myelin water T_2_ is defined as the T_2_ times of the distribution between 8 ms and 25 ms. The intracellular and extracellular water T_2_ is defined as the T_2_ times above 25 ms. The cutoff between the two water pools was set to 25 ms, which was based on the measured T_2_ distributions. The apparent MWF was calculated as the ratio of the myelin water T_2_ to the entire T_2_ components.

The T_1_ and DTI was linearly registered to the MWI using FLIRT. FSL’s vecreg was used to register the first eigenvector to the MWI space. The fiber orientation was calculated from the angle between the first eigenvector and the direction of the main magnetic field B_0_.^21^ Brain extraction was performed using FSL’s brain extraction tool (bet), and FSL’s FAST was used to generate a white matter mask, which was eroded with a 3 × 3 × 3 kernel. The apparent MWF, myelin water T_2_, and intracellular and extracellular water T_2_ were computed as a function of local fiber orientation *θ*. The fiber orientations were divided into 18 intervals of 5° between 0° (parallel to B_0_) and 90° (perpendicular to B_0_). To compute apparent MWF (*θ*), voxels from across the entire white matter were pooled for each angle interval, to reduce the influence of tract‐specific differences in apparent MWF.

### 2.3 Statistical analysis

All parameters were tested for normal distribution using the Shapiro‐Wilk test. A t‐test was used to determine the significance of differences between GRASE and CPMG. To test whether the fiber angle and TR had a significant effect on apparent MWF and T_2_, an analysis of variance was used. All statistical analyses were performed using R (version 3.5.1; The R Foundation for Statistical Computing).

## 3 RESULTS

**FIGURE 1:**
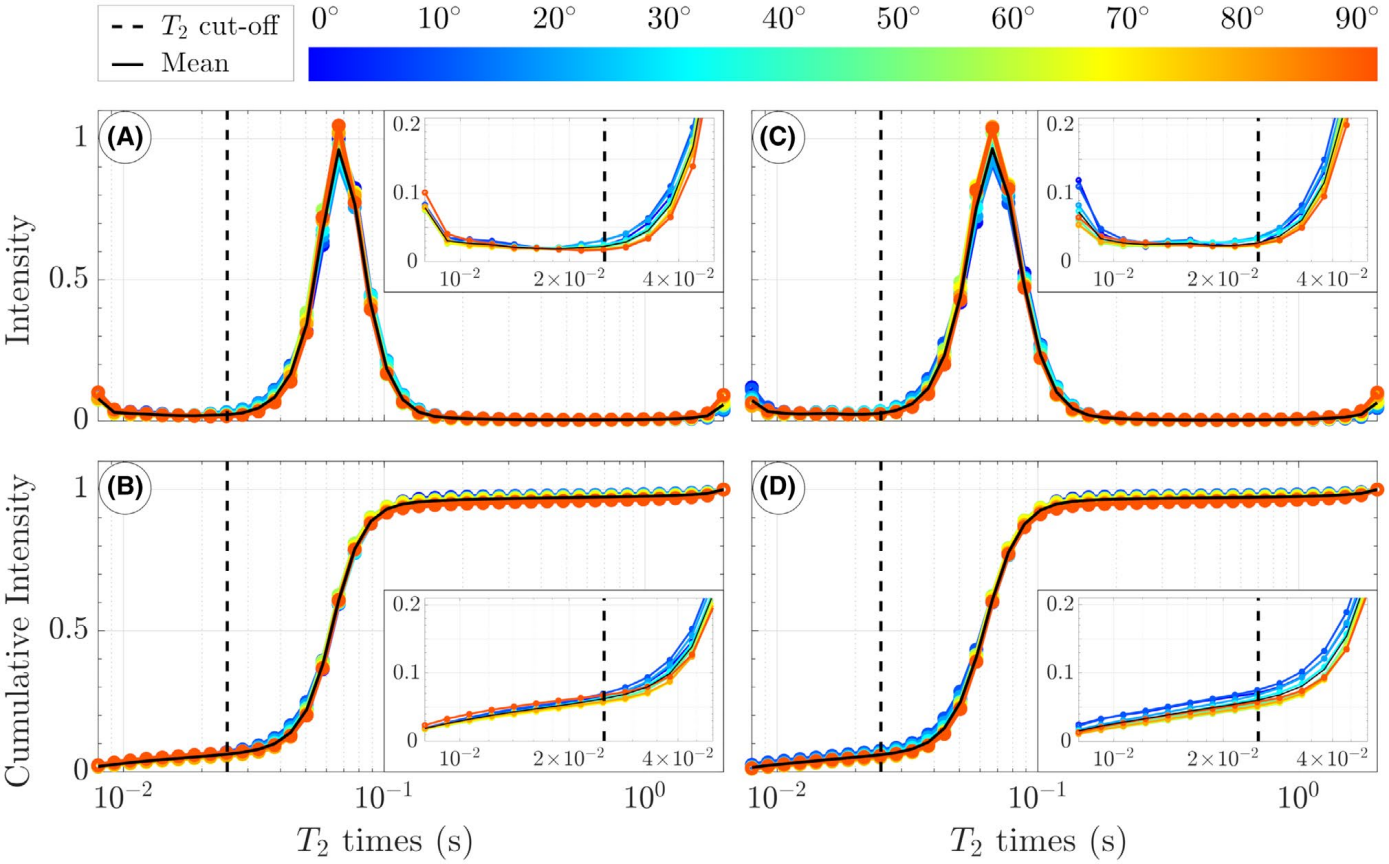
T_2_ distribution of a representative subject at different fiber angle intervals for Carr‐Purcell‐Meiboom‐Gill (CPMG) (A) and gradient and spin echo (GRASE) (C). Please note that the distribution is dominated by the intracellular and extracellular water peak. The myelin water peak and the cerebrospinal fluid peak are represented by increased signal intensity at the left and right border of the plot, respectively. In the lower row, the cumulative signal intensity is plotted as function of T_2_ times for CPMG (B) and GRASE (D). The zoomed inserts in (B) and (D) show how the integral is maximal for low angles and reaches a minimum for angles approximately 50° to 60°. The vertical dashed line indicates the cutoff of 25 ms used in the present study. The typically used cutoff of 40 ms results in a larger orientation dependency in apparent myelin water fraction (MWF)

The T_2_ distributions plotted for different fiber angles of a representative subject are shown for CPMG in Figure 1A and for GRASE in Figure 1C. The distributions exhibit the typical large peak from intracellular and extracellular water, the small myelin water peak at short T_2_ relaxation times, and a small peak from CSF at very long T_2_ relaxation times. The plotted T_2_ distribution highlights the intracellular and extracellular water peak, whereas the myelin water peak and CSF peak are represented by increased signal intensity at the left and right border of the plot, respectively. The zoomed inserts display the systematic angle dependency of the distribution. Figure 1B,D shows the cumulative intensity of the distribution ranging from T_2,min_ = 8 ms to T_2_ = T_2,max_, computed as the integral of the area under the T_2_ distribution from T_2,min_ to the respective T_2_ time normalized by the total area of the T_2_ distribution. The zoomed insert shows how the integral differs between fiber angles and between the CPMG and GRASE sequence.

**FIGURE 2:**
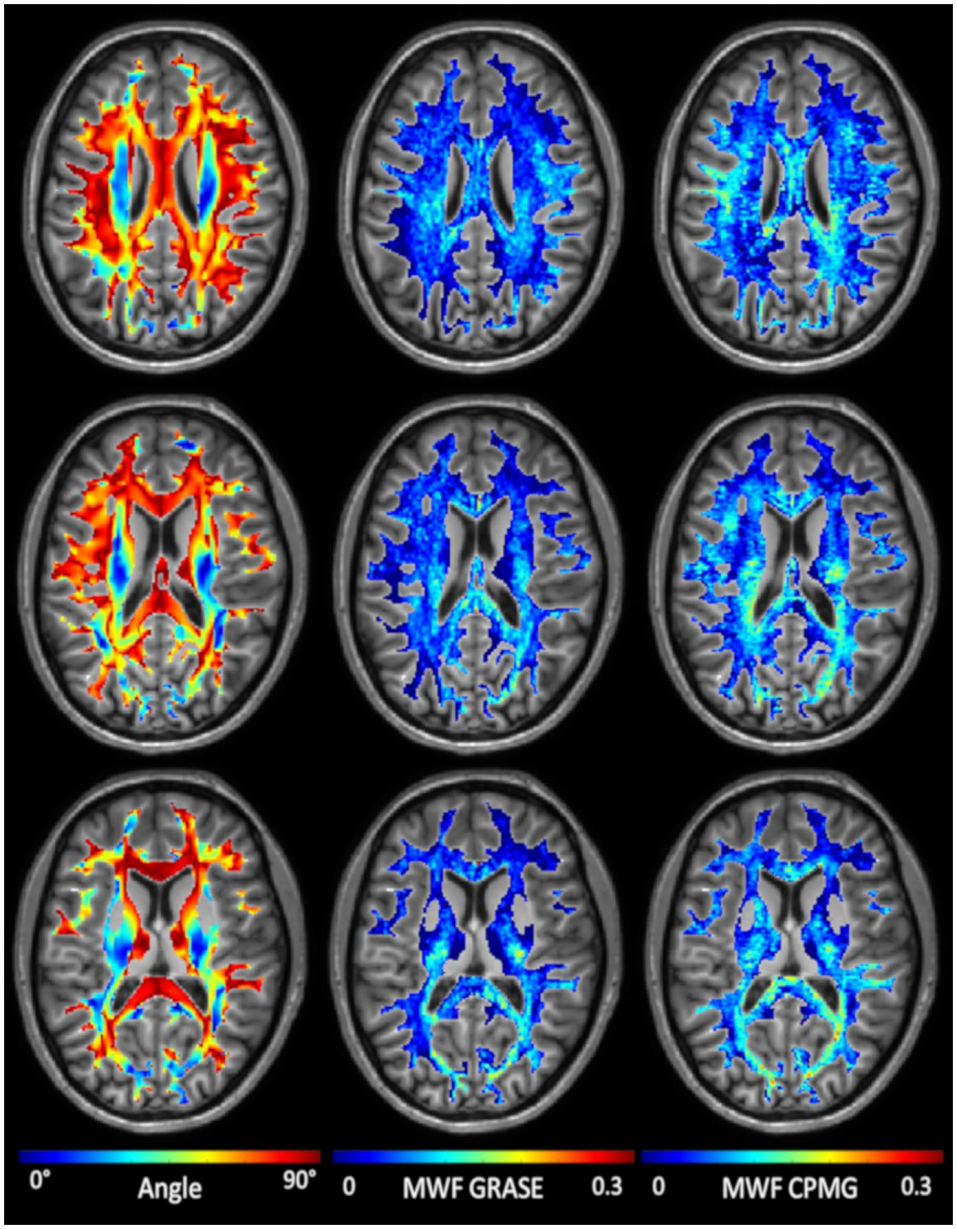
Representative fiber angle and apparent MWF maps, acquired using a CPMG and GRASE sequence, of a single subject

Representative maps of the angle between white matter fibers and B_0_ and the corresponding apparent MWF maps are shown in Figure 2 for a single subject. Overall, the GRASE and CPMG sequences provide comparable results, although using a CPMG sequence resulted in higher apparent MWF.

**FIGURE 3:**
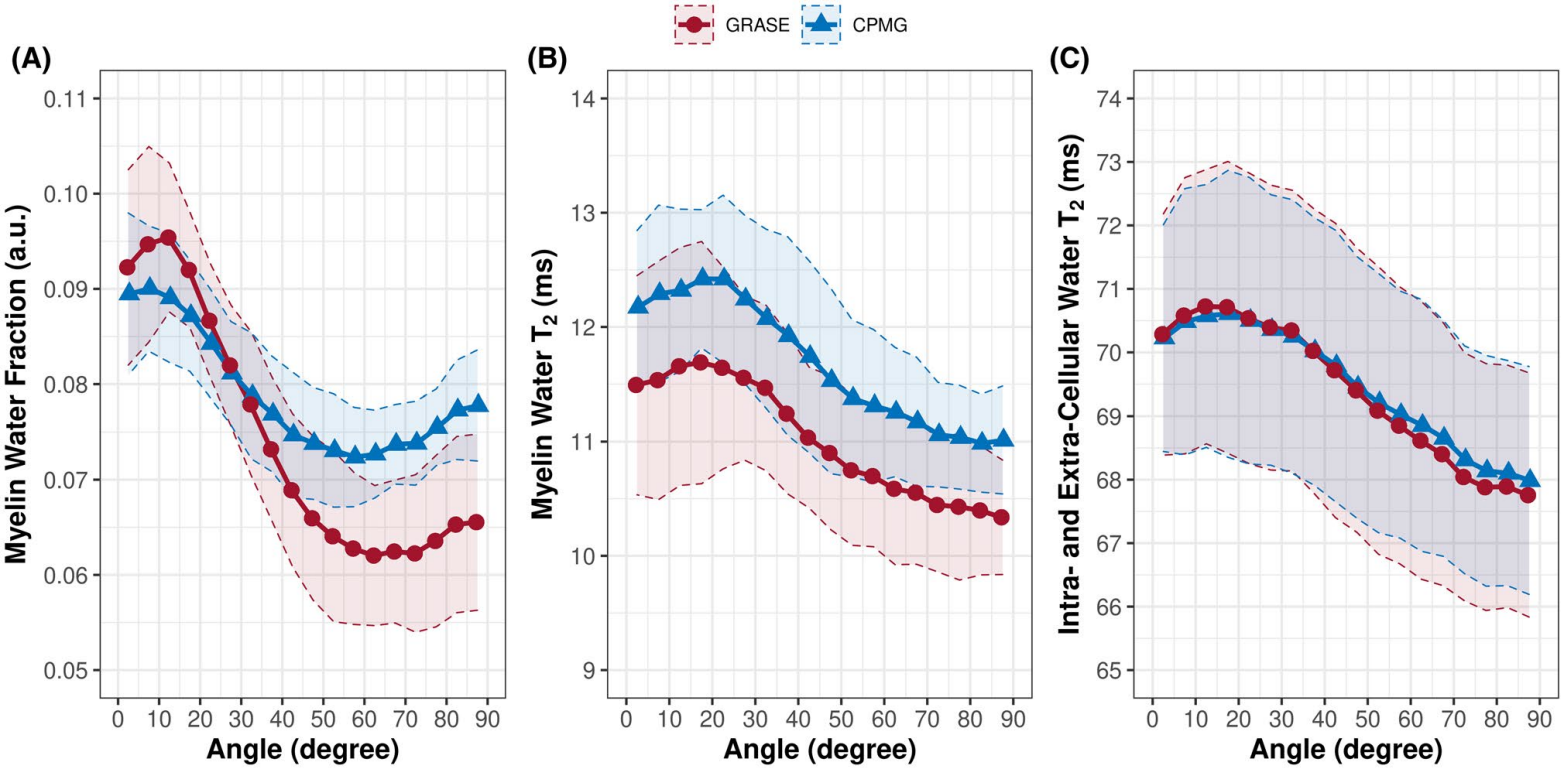
Apparent MWF (A), myelin water T_2_ (B), and intracellular and extracellular water T_2_ (C) as function of fiber orientation acquired using a GRASE (red, 7 subjects) and CPMG (blue, 8 subjects) sequence, both with TR = 1073 ms. The shaded areas indicate the 95% confidence interval

The relationship among the apparent MWF, myelin water T_2_, intracellular and extracellular water T_2_, and the white matter fiber orientation is shown in Figure 3. In general, the apparent MWF decreased with increasing fiber angles and reached a minimum between 50° and 60°, followed by an increase toward angles of 90° (Figure 3A). The apparent MWF varied by approximately 35% for the GRASE sequence, and by approximately 22% for the CPMG sequence. Using a GRASE sequence resulted in a stronger orientation dependency of the apparent MWF. Myelin water T_2_ (Figure 3B) and intracellular and extracellular water T_2_ (Figure 3C) increased with increasing fiber angle up to a maximum between 15° and 25° followed by a decrease. Between GRASE and CPMG, there was a significant difference in the orientation dependency of the apparent MWF (*P* = .002) and the myelin water T_2_ (*P* < .001). The orientation dependency of the intracellular and extracellular water T_2_ (*P* = .79) showed no significant difference between the two scan types.

### 3.1 Effect of TR

**FIGURE 4:**
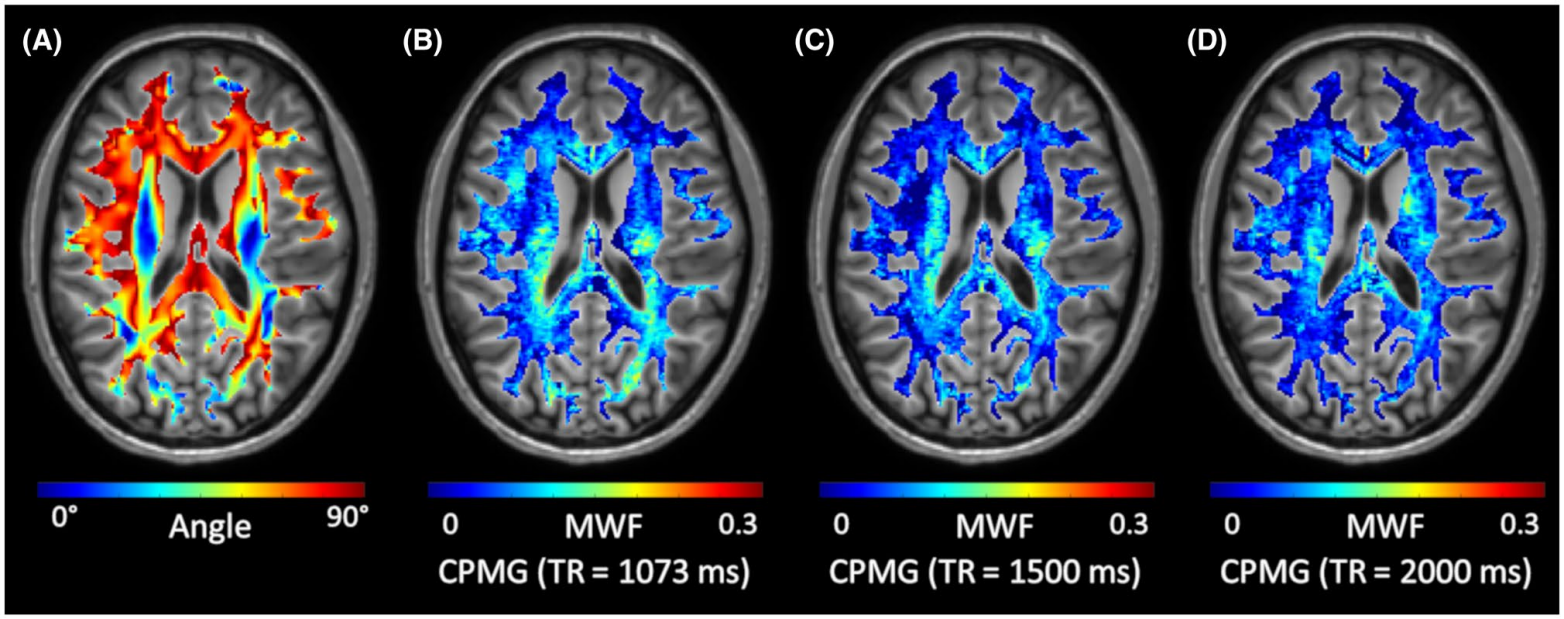
Representative fiber angle and apparent MWF maps of a single subject acquired using a CPMG sequence with TR = 1073 ms, 1500 ms and 2000 ms. Overall, apparent MWF decreases with increasing TR

**FIGURE 5:**
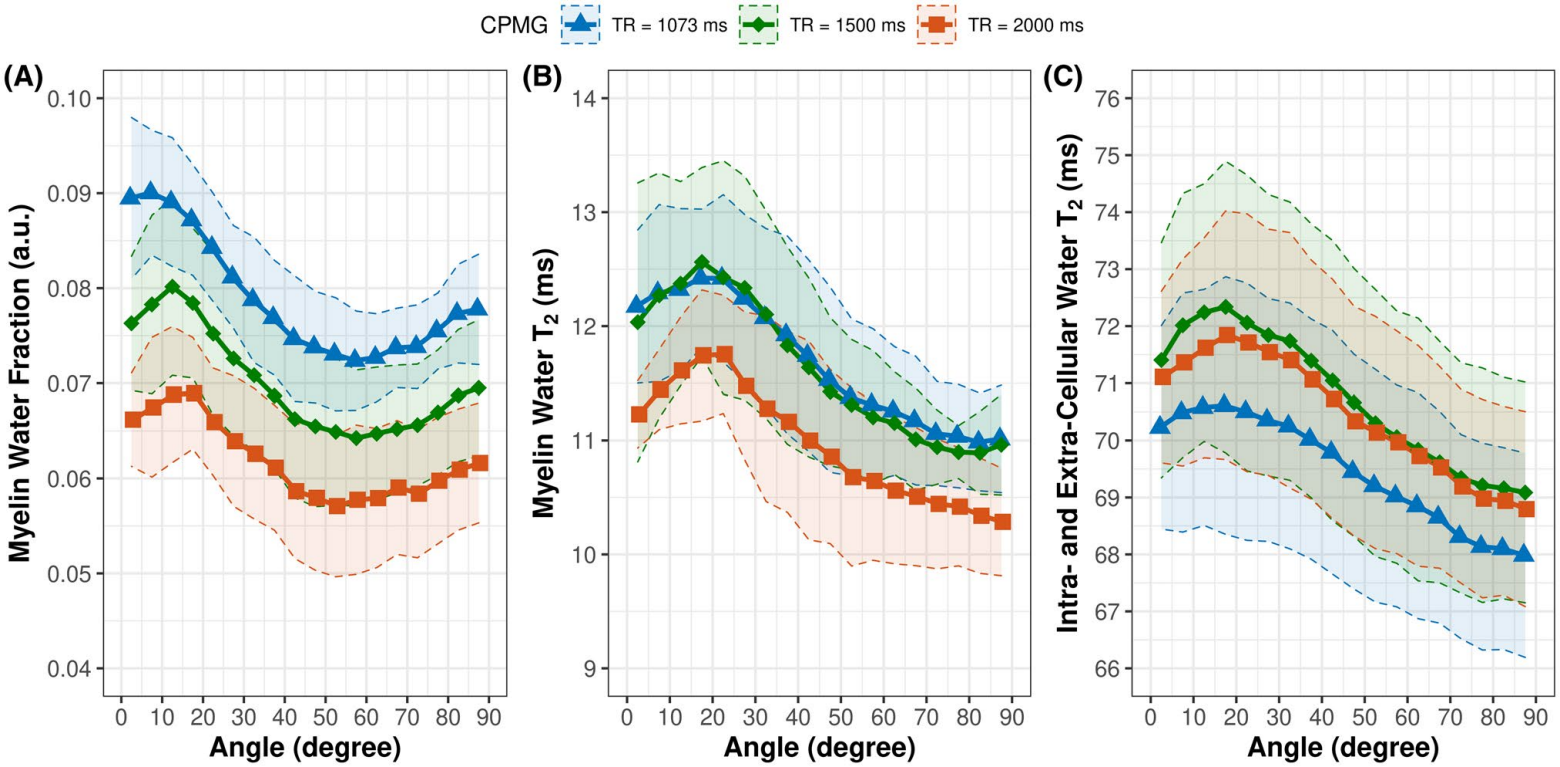
Apparent MWF (A), myelin water T_2_ (B), and intracellular and extracellular water T_2_ (C) as function of fiber orientation acquired using a CPMG sequence with a TR of 1073 ms (blue, 8 subjects), 1500 ms (green, 6 subjects), and 2000 ms (orange, 4 subjects). The minimum of the orientation‐dependent apparent MWF was between 50° and 60° for all TRs. The shaded areas indicate the 95% confidence interval

**FIGURE 6:**
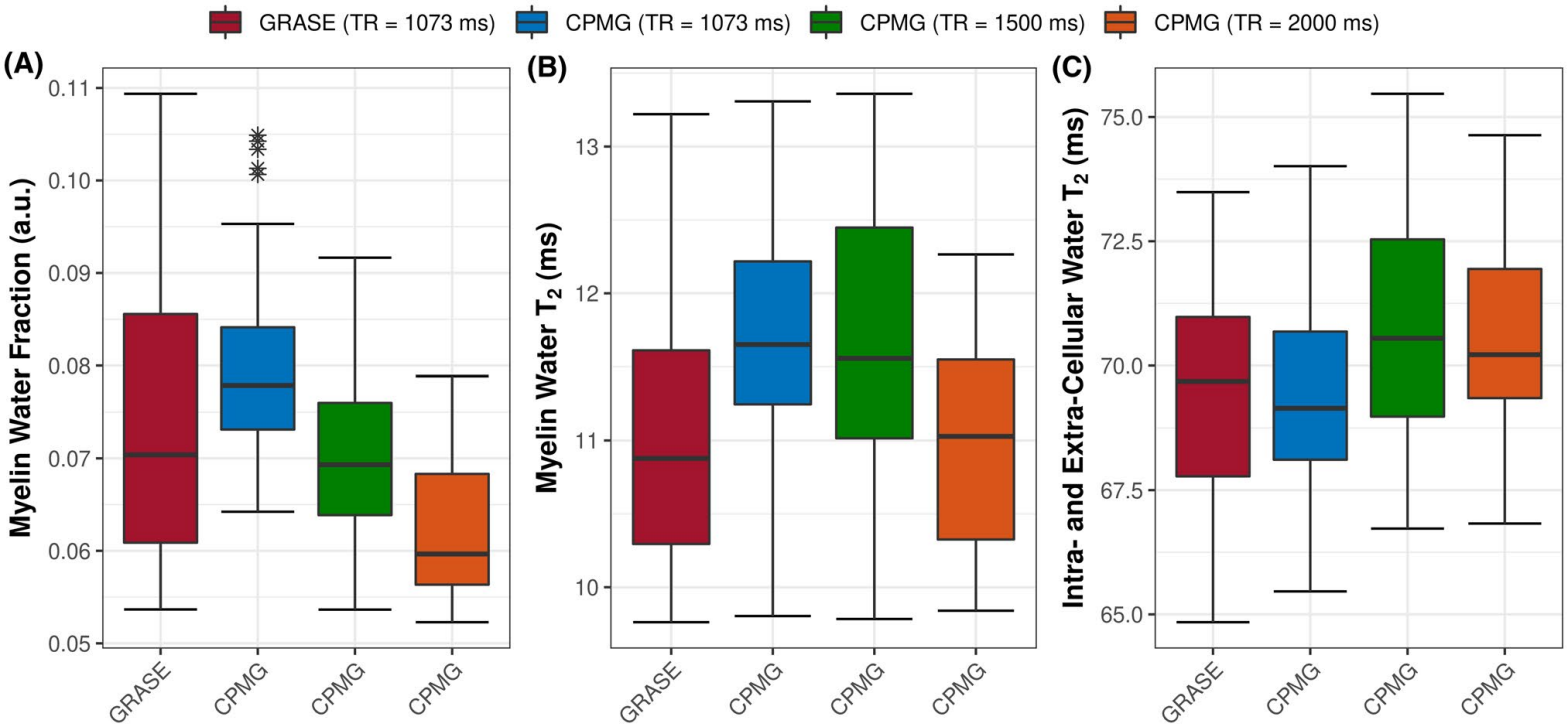
Global white matter apparent MWF (A), myelin water T_2_ (B), and intracellular and extracellular water T_2_ (C). The TR has a significant impact on the apparent MWF (*P* < .001), myelin water T_2_ (*P* < .001), and intracellular and extracellular water T_2_ (*P* < .001). The GRASE sequence was acquired on 7 subjects; CPMG with TR of 1073 ms was acquired on all 8 subjects, with TR of 1500 ms on 6 subjects and TR of 2000 ms on 4 subjects

By increasing TR from 1073 ms to 1500 ms and 2000 ms, the orientation‐dependent apparent MWF decreased across all regions, as shown in Figure 4. In general, TR had a significant influence on the orientation‐dependent apparent MWF (*P* < .001), myelin water T_2_ (*P* < .001), and intracellular and extracellular water T_2_ (*P* < .001), as shown in Figure 5. The shape of the orientation‐dependent apparent MWF, myelin water T_2_, and intracellular and extracellular water T_2_ was similar but shifted with increasing TR. The effect of TR is further evident by plotting the global white matter mean of the apparent MWF (Figure 6A), myelin water T_2_ (Figure 6B), and intracellular and extracellular water T_2_ (Figure 6C). For example, the apparent MWF decreased by 21.5% from 0.079 at TR = 1073 ms to 0.062 at TR = 2000 ms (*P* = .006), as shown in Figure 6A.

## 4 DISCUSSION

We demonstrated that the T_2_ relaxation times of both myelin water and intracellular and extracellular water, and therefore the measurement of the apparent MWF, are considerably influenced by the angle between the white matter fiber tracts and the main magnetic field. Furthermore, the TR of the sequence also has an impact on estimating the apparent MWF. Orientation effects of the transverse relaxation signal have been ascribed primarily to dipole–dipole interaction and to magnetic susceptibility effects. In the following, we first discuss both mechanisms qualitatively in the context of previous literature. Then, we apply previously presented models to our experimental data to investigate the possible origins of the orientation dependency.

The observed orientation dependency of the apparent MWF is based on the fact that the underlying T_2_ distribution depends on the fiber angle. As the estimation of the apparent MWF relies on the separation of the myelin water pool from the intracellular and extracellular water pool in the T_2_ spectrum, an alteration of the T_2_ spectrum will affect the estimation of the apparent MWF. This is further evident as the myelin water pool and the intracellular and extracellular water pool show a different sensitivity to changes in fiber orientation. Furthermore, as the T_2_ spectrum is based on the T_2_ relaxation properties per se, the orientation‐dependent behavior of the apparent MWF can be related through the orientation dependency of the T_2_ spectrum to T_2_ anisotropy effects in general.

Orientation dependency of T_2_ has been observed in highly ordered tissues, such as cartilage and tendons.^48^, ^49^, ^50^, ^51^ Such structures give high T_2_ signal when they are oriented near the magic angles, where the term 3 cos^2^ *θ* − 1 describing the z‐component of the dipole field approaches zero. When water is bound to highly ordered tissue, the dipolar interactions depend on the orientation of the tissue with respect to the main magnetic field. These dipolar interactions result in a rapid signal dephasing, unless the tissue is oriented at or near the magic angle. In experimental work on tendons,^50^, ^52^ where signal increases by several 100% are observed at the magic angle, the orientation‐dependent relaxation effects could be modeled according to k·(3 cos^2^ *θ* − 1)^2^.^48^, ^51^

Another cause of orientation effects is the magnetic susceptibility of tissue, which has a strong influence on phase and magnitude of the gradient‐echo signal. Although the orientation dependency of white matter $R_{2}^{*}$ has been studied extensively,^20^, ^21^, ^22^, ^24^, ^25^, ^53^ the effect of tissue orientation on R_2_ in the brain has been considered weak for a long time.^37^, ^49^ One notable exception is diffusion in the vicinity of the anisotropic white matter vasculature, which gives rise to considerable orientation dependency in the measurements of cerebral blood flow and volume using spin‐echo dynamic susceptibility contrast.^33^ Oh et al reported a weak orientation dependency of R_2_ in ex vivo formalin‐fixed white matter that is best described by dipole–dipole interaction according to k·(3 cos^2^ *θ* − 1)^2^.^25^ Based on their observation, the authors suggested that the apparent MWF should also weakly depend on fiber orientation. However, the angle effects in their R_2_ data were small, which could be a consequence of fixation‐induced cross‐linking, which reduces tissue anisotropy at length scales that are relevant to T_2_ relaxation. Furthermore, with the decreased T_2_ relaxation times due to tissue fixation^54^ and due to the high field strength of 7 T, the shortest TE of 9 ms of their study may be too long to capture orientation effects under these conditions. Formalin fixation also reduces the diffusion coefficient, and therefore the effects of magnetic susceptibility on the spin‐echo signal. The orientation dependency of the field inhomogeneities, on the contrary, is not changed by tissue fixation, as demonstrated by various studies on $R_{2}^{*}$ in fixed tissue.^25^

Knight et al presented a model for the orientation dependency of the spin‐echo signal based on the loss of spin coherence due to diffusion through field inhomogeneities.^36^, ^37^ This loss of coherence adds to the intrinsic R_2_ and results in an additional reduction of the spin‐echo signal. For anisotropic perturbers, such as cylindrical structures, the spin‐echo signal becomes orientation‐dependent. The theory predicts two possible sources of T_2_ anisotropy, which are referred to as (1) a sin^4^ (*θ*)‐related orientation dependency caused by diffusion‐mediated loss of coherence due to susceptibility differences and (2) a sin^2^ (*θ*) orientation dependency related to interactions between susceptibility differences and applied field gradients.^37^ Experimentally, Knight et al report the longest T_2_ times for white matter fibers parallel to the B_0_, and the shortest T_2_ times for white matter fibers perpendicular to B_0_, which is in good agreement with their theoretical framework. These results are also in agreement with our present study, in which we observed the longest T_2_ times of both myelin water T_2_ and intracellular extracellular water T_2_ at low angles, and the shortest T_2_ times in tissue perpendicular to the main magnetic field. Note, however, that in our data both the myelin water T_2_ and the intracellular and extracellular water T_2_ exhibit a small increase between 0° and about 20°, which is not explained by the model by Knight et al.

**FIGURE 7:**
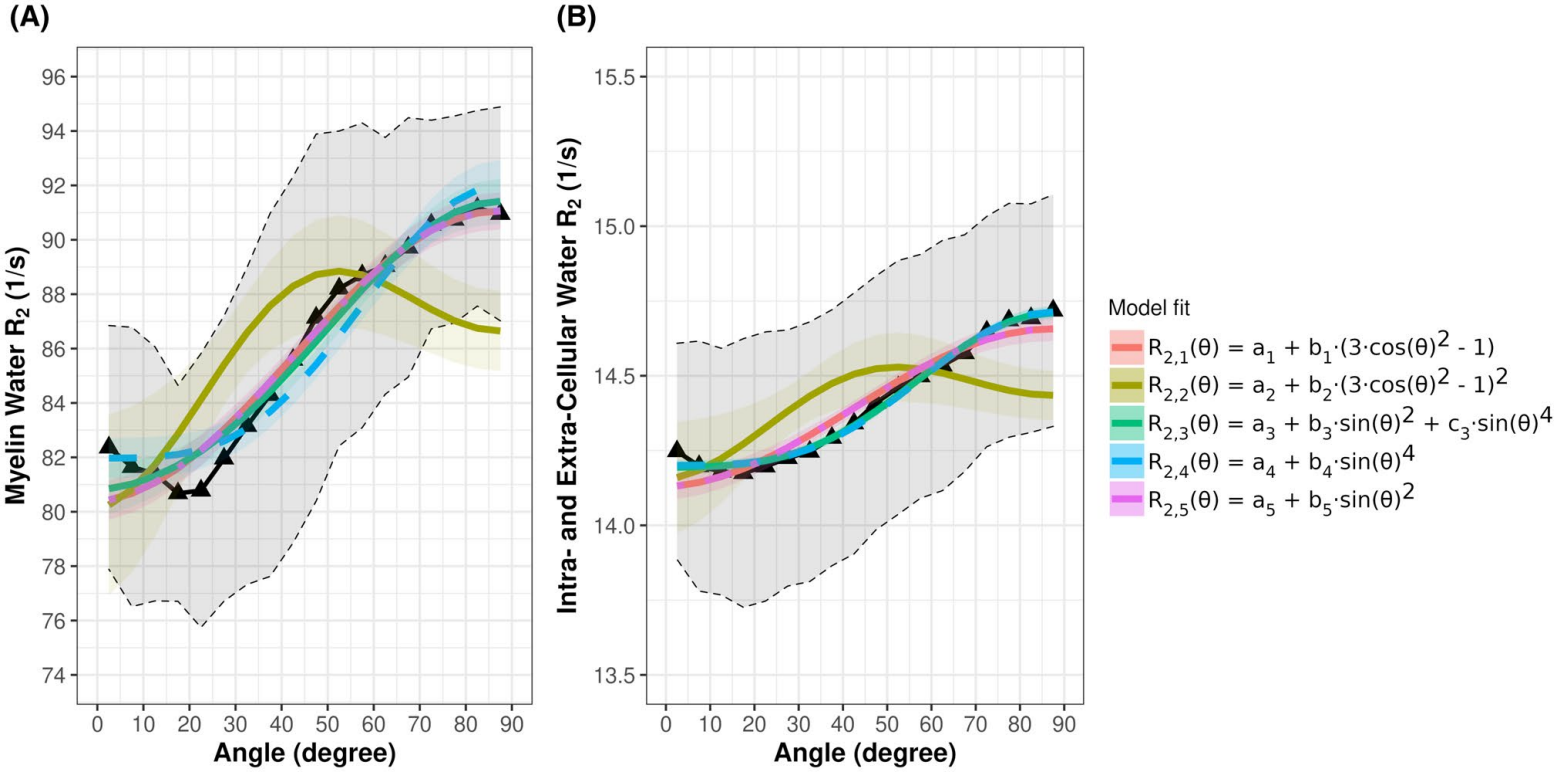
Different models proposed in literature describing orientation‐dependent effects were fitted to the measured myelin water R_2_ and intracellular and extracellular water *R*_2_, acquired using a CPMG sequence with TR = 1073 ms (black curve). The shaded area represents the 95% confidence interval. All fit parameters are summarized in Supporting Information Tables S2 and S3

To shed more light on the underpinnings of the orientation dependency of the spin‐echo signal, we fitted previously presented models to our measured myelin water R_2_ and intracellular and extracellular water *R*_2_ data (Figure 7 and Supporting Information Tables S2 and S3).

The classical dipole interaction model^51^, ^55^ with an orientation‐independent contribution a_1_ is

(1) $$R_{2,1}\left(\theta \right)=a_{1}+b_{1}\;\cdot \;\left(3\cos^{2}\left(\theta \right)‐1\right),$$

which allows us to fit the measured myelin water R_2_ and the intracellular and extracellular water R_2_ (Figure 7). In the next step, we use the extended dipole–dipole interaction model according to previous studies,^25^, ^48^, ^52^ in which the model was used to fit orientation‐dependent R_2_ according to

(2) $$R_{2,2}\left(\theta \right)=a_{2}+b_{2}\;\cdot \;{\left(3\cos^{2}\left(\theta \right)‐1\right)}^{2}.$$

Fitting model *R*_2,2_ (*θ*) resulted in a poor fit of the R_2_ signal (Figure 7).

The third model S_3_ (*θ*) was based on the theory that Knight et al^37^ developed for describing the influence of magnetic susceptibility on the orientation‐dependent R_2_ signal according to

(3) $$R_{2,3}\left(\theta \right)=a_{3}+b_{3}\;\cdot \;\sin^{2}\left(\theta \right)+c_{3}\;\cdot \;\sin^{4}\left(\theta \right).$$

Orientation effects due to interactions between susceptibility differences and applied field gradients contribute to *b*_3_·sin^2^ (*θ*), and orientation effects due to diffusion‐mediated loss of coherence contribute to *c*_3_·sin^4^ (*θ*). This model allowed us to properly fit the myelin water R_2_ and intracellular and extracellular water R_2_ (Figure 7).

In addition, if we independently investigate the orientation effects relating to sin^4^ (*θ*) and contributions related to sin^2^ (*θ*), we see that this allows us to fit the myelin water R_2_ and intracellular and extracellular water R_2_ (Figure 7). It should be noted that dipole–dipole interactions expressed by *R*_2,1_ (*θ*) = *a*_1_ + *b*_1_ (3 cos^2^ (*θ*) − 1) can be rewritten as *R*_2,1_ (*θ*) = *a*_1_ + 2b_1_ − 3*b*_1_ sin^2^ (*θ*), which is also evident as the curves of both model fits superimpose, as shown in Figure 7. Furthermore, model *R*_2,2_ (*θ*) (Equation 2) can be rewritten as *R*_2,2_ (*θ*) = a_2_ + 4*b*_2_ − 12*b*_2_ sin^2^ *θ* + 9*b*_2_ sin^4^ *θ*, which is in line with model R_2,3_ (*θ*), where *a*_3_ = *a*_2_ + 4*b*_2_, *b*_3_ = −12*b*_2_ and *c*_3_ = 9*b*_2_. This supposes that model R_2,3_ (*θ*) also incorporates orientation effects due to dipole–dipole interaction, along with orientation effects due to magnetic susceptibility effects. The fitting parameters of all different models are summarized in Supporting Information Table S2 for myelin water *R*_2_, and in Supporting Information Table S3 for intracellular and extracellular water R_2_.

Other contributions, such as T_1_ relaxation and magnetization transfer, will affect the different T_2_ components, and therefore the estimation of the apparent MWF. The dependency of apparent MWF and T_2_ on TR could be additionally affected by the different T_1_ relaxation times of myelin water and the intracellular and extracellular water. The overall T_1_ of white matter at 3 T is about 1100 ms,^56^, ^57^ whereas T_1_ of myelin water is about 200 ms.^58^, ^59^, ^60^ Therefore, myelin water is almost fully relaxed even at the shortest TR used in the present study, whereas the intracellular and extracellular water signal is attenuated and increases with increasing TR. As a result, the apparent MWF decreases with increasing TR. Schyboll et al showed a small orientation dependency in both T_1_ and water content measurements, of 2.5% and 0.8%, respectively.^28^, ^31^ Interestingly, the shape of the water content curve mirrors the apparent MWF curve in our study. In a very recent paper, the same authors showed that the susceptibility effects of the myelin sheath only cause a very weak orientation dependency in T_1_ of about 0.4 · 10^−4^ Hz between parallel fibers and fibers at the magic angle.^29^ Furthermore, Knight et al also reported that the T_1_ of white matter weakly depends on the fiber angle with the highest T_1_ near the magic angle of 54.7°.^30^

Finally, magnetization‐transfer effects may influence the estimation of the apparent MWF. Pampel et al showed that magnetization‐transfer experiments in white matter are sensitive to fiber‐orientation effects, and therefore affect the estimation of short T_2_ components through the fiber orientation dependency of the RF absorption lineshape. In their experiments, they observed a fiber orientation–dependent T_2_ of the bound water pool (T_2,b_), with a similar orientation dependency compared to our observed myelin water T_2_. The orientation dependency in T_2,b_ was attributed to chemical exchange and cross‐relaxation effects.^61^

One limitation of the study is that we only investigated TR up to 2000 ms, which is approximately two times the T_1_ of the long T_1_ component. The shortest TR of 1073 ms, on the contrary, was defined by the specific absorption rate of the scan. With the required anatomical T_1_ and DTI scans and the comparison with the GRASE approach at 1073 ms, the total scan protocol took approximately 75 minutes. Therefore, the present study is not able answer the question of how apparent MWF behaves in the absence of T_1_ weighting. A comprehensive study that explores the effects of TR would require several long scans with TRs of up to 5000 ms, in which T_1_ weighting of the intracellular and extracellular water is reduced to less than 2%. Such study may be feasible by not scanning every TR in every participant and with reduced brain coverage and lower spatial resolution, which both make image registration more challenging.

Furthermore, the orientation dependency of GRASE and CPMG were different, particularly at larger angles. The increase in apparent MWF at angles above 54.7° was stronger for CPMG compared with GRASE, which may be due to some $R_{2}^{*}$ weighting of the GRASE sequence, leading to a reduction of the signal due to field inhomogeneities created by the myelinated axons at larger angles. Future MWI studies will increasingly use the CPMG sequence with compressed sensing, as it allows for shorter echo spacing and for shorter scan times compared with the GRASE‐based approach.^43^

The approach of using DTI to determine the fiber orientation and pooling voxels according to their local fiber orientation was previously applied in several studies.^20^, ^21^, ^24^, ^32^, ^62^ Because for each angle interval voxels are pooled from across the entire brain, potential confounding effects of tract specific differences in apparent MWF are reduced. Moreover, apparent MWF (*θ*) of all subjects followed a similar pattern, independently of individual differences in head orientation or white matter anatomy. According to Knight et al, crossing fibers led to a decrease in T_2_ compared with voxels containing only a single fiber orientation, which will affect the observed orientation‐dependent T_2_, and therefore the estimated apparent MWF.^63^ By using high‐resolution DTI acquisitions with high b‐values and multiple directions, the influence of crossing fibers can be minimized.^19^

Our findings have consequences for the interpretation of past results and for future research. The orientation dependency of the apparent MWF might mask longitudinal changes in MWF or changes between cohorts, if whole white matter averages are investigated. A corresponding effect was observed in white matter $R_{2}^{*}$, where only an orientation‐dependent analysis was able to reveal group differences.^53^, ^62^ Kor et al modeled the $R_{2}^{*}$ relaxation as a function of white matter orientation, myelin, and iron content, and were able to compute whole white matter myelin and iron content.^53^ This approach assumed that the effects of iron are orientation‐independent, and that only the myelin sheath gives rise to an orientation‐dependent $R_{2}^{*}$. Because variations in iron concentration explain approximately 25% of the measured variance in the apparent MWF,^64^ an orientation‐dependent analysis accompanied by a model that includes the effects of both myelin and iron may shed further light on the orientation dependency of the apparent MWF.

Our findings suggest that comparison of apparent MWF of individual subjects may exhibit paradoxical results if their head orientation differs. A difference in angle by 10°, for example, would result in a relative difference in apparent MWF by 5% to 7%, which is similar to the reduction in apparent MWF in the normal‐appearing white matter in multiple sclerosis over 5 years.^17^

In conclusion, our study shows that the T_2_ distribution, myelin water T_2_, and the intracellular and extracellular water T_2_, as well as the apparent MWF, depend on white matter fiber orientation. Furthermore, with increasing TR, the overall apparent MWF decreases, while the orientation dependency persists.

## Supporting information


**TABLE S1** Summary of all volunteers, the corresponding acquired sequences, and the acquisition time of each sequence
**TABLE S2** Fitting parameters of the different models to the measured myelin water R_2_ data
**TABLE S3** Fitting parameters of the different models to the measured intracellular and extracellular water R_2_
