# Supplementary material for: Myelin water imaging depends on white matter fiber orientation in the human brain
Source: Magn Reson Med. 2020 Oct 5;85(4):2221–31. doi: 10.1002/mrm.28543 (PMC7821018; doi:10.1002/mrm.28543)
Supplement: Supplementary file 1 — TABLE S1 Summary of all volunteers, the corresponding acquired sequences, and the acquisition time of each sequence TABLE S2 Fitting parameters of the different models to the measured myelin water R2 data TABLE S3 Fitting parameters of the different models to the measured intracellular and extracellular water R2 [file MRM-85-2221-s001.docx]

**Supporting Information**

**Myelin water imaging depends on white matter fiber orientation in the human brain**

**Supporting Information Table S1:** Summary of all volunteers, the corresponding acquired sequences, and the acquisition time of each sequence.

|  |  | **Age** | **T1** | **DTI** | **GRASE** | **CPMG** | **CPMG TR = 1500 ms** | **CPMG TR = 2000 ms** |
| --- | --- | --- | --- | --- | --- | --- | --- | --- |
| **Volunteer** | **Sex** | (years) | 5:50 min | 4:19 min | 11:29 min | 09:43 min | 13:33 min | 18:04 min |
| 1 | M | 29 | x | x | x | x |  |  |
| 2 | M | 26 | x | x | x | x | x |  |
| 3 | F | 31 | x | x | x | x | x | x |
| 4 | M | 33 | x | x | x | x | x |  |
| 5 | F | 22 | x | x | x | x | x |  |
| 6 | M | 23 | x | x | x | x | x | x |
| 7 | F | 22 | x | x |  | x |  | x |
| 8 | M | 21 | x | x | x | x | x | x |

**Supporting Information Table S2:** Fitting parameters of the different models to the measured myelin water R_2_ data.

| **Model** | **Sequence** | **TR (ms)** | **a** | **95% CI** | **b** | **95% CI** | **c** | **95% CI** | **RSE** |
| --- | --- | --- | --- | --- | --- | --- | --- | --- | --- |
| R_2,1_(θ) | GRASE | 1073 | 92.78 | (92.28 – 93.27) | -3.67 | (-4.11 - -3.24) | - | - | 0.923 |
|  | CPMG | 1073 | 87.49 | (87.05 – 87.93) | -3.57 | (-3.96 - -3.19) | - | - | 0.817 |
|  |  | 1500 | 88.10 | (87.42 – 88.76) | -3.82 | (-4.40 - -3.23) | - | - | 1.242 |
|  |  | 2000 | 93.12 | (92.45 – 93.79) | -3.70 | (-4.29 - -3.11) | - | - | 1.247 |
| R_2,2_(θ) | GRASE | 1073 | 94.04 | (91.82 – 96.27 | -2.16 | (-3.40 - -0.93) | - | - | 3.094 |
|  | CPMG | 1073 | 88.85 | (86.79 – 90.90) | -2.20 | (-3.34 - -1.06) | - | - | 2.864 |
|  |  | 1500 | 89.49 | (87.16 – 91.81) | -2.31 | (-3.60 - -1.01) | - | - | 3.237 |
|  |  | 2000 | 94.47 | (92.20 – 96.74) | -2.24 | (-3.50 - -0.98) | - | - | 3.159 |
| R_2,3_(θ) | GRASE | 1073 | 86.20 | (85.24 – 87.15) | 5.40 | (0.59 – 10.21) | 5.46 | (0.92 – 10.00) | 0.795 |
|  | CPMG | 1073 | 80.79 | (79.84 – 81.74) | 7.47 | (2.71 – 12.23) | 3.16 | (-1.35 – 7.66) | 0.787 |
|  |  | 1500 | 81.07 | (79.60 – 82.53) | 7.02 | (-0.32 – 14.36) | 4.31 | (-2.63 – 11.25) | 1.213 |
|  |  | 2000 | 86.30 | (84.83 – 87.78) | 6.78 | (-0.62 – 14.17 | 4.21 | (-2.78 – 11.21) | 1.222 |
| R_2,4_(θ) | GRASE | 1073 | 87.05 | (86.39 – 87.72) | 10.42 | (9.21 – 11.63) | - | - | 0.905 |
|  | CPMG | 1073 | 81.97 | (81.23 – 82.71) | 10.02 | (8.67 – 11.37) | - | - | 1.007 |
|  |  | 1500 | 82.18 | (81.20 – 83.15) | 10.75 | (8.98 – 12.53) | - | - | 1.328 |
|  |  | 2000 | 87.37 | (86.40 – 88.35) | 10.44 | (8.66 – 12.22) | - | - | 1.325 |
| R_2,5_(θ) | GRASE | 1073 | 85.44 | (84.61 – 86.27) | 11.01 | (9.71 – 12.32) | - | - | 0.923 |
|  | CPMG | 1073 | 80.35 | (79.61 – 81.08) | 10.72 | (9.56 – 11.87) | - | - | 0.817 |
|  |  | 1500 | 80.47 | (79.35 – 81.58) | 11.45 | (9.69 – 13.21) | - | - | 1.242 |
|  |  | 2000 | 85.71 | (84.59 – 86.84) | 11.11 | (9.34 – 12.88) | - | - | 1.247 |

**Supporting Information Table S3:** Fitting parameters of the different models to the measured intra- and extra-cellular water R_2_ data.

| **Model** | **Sequence** | **TR (ms)** | **a** | **95% CI** | **b** | **95% CI** | **c** | **95% CI** | **RSE** |
| --- | --- | --- | --- | --- | --- | --- | --- | --- | --- |
| R_2,1_(θ) | GRASE | 1073 | 14.51 | (14.48 – 14.54) | -0.20 | (-0.23 - -0.18) | - | - | 0.053 |
|  | CPMG | 1073 | 14.48 | (14.45 – 14.51) | -0.18 | (-0.20 - -0.15) | - | - | 0.049 |
|  |  | 1500 | 14.24 | (14.21 – 14.27) | -0.21 | (-0.24 - -0.18) | - | - | 0.062 |
|  |  | 2000 | 14.29 | (14.25 – 14.32) | -0.19 | (-0.22 - -0.16) | - | - | 0.064 |
| R_2,2_(θ) | GRASE | 1073 | 14.57 | (14.44 – 14.70) | -0.11 | (-0.18 - -0.04) | - | - | 0.183 |
|  | CPMG | 1073 | 14.53 | (14.41 – 14.64) | -0.09 | (-0.16 - -0.03) | - | - | 0.160 |
|  |  | 1500 | 14.30 | (14.17 – 14.44) | -0.12 | (-0.19 - -0.04) | - | - | 0.187 |
|  |  | 2000 | 14.33 | (14.21 – 14.46) | -0.10 | (-0.17 - -0.03) | - | - | 0.178 |
| R_2,3_(θ) | GRASE | 1073 | 14.17 | (14.14 – 14.20) | 0.09 | (-0.06 – 0.25) | 0.51 | (0.36 – 0.65) | 0.025 |
|  | CPMG | 1073 | 14.19 | (14.17 – 14.22) | 0.04 | (-0.09 – 0.18) | 0.47 | (0.35 – 0.60) | 0.022 |
|  |  | 1500 | 13.89 | (13.83 – 13.94) | 0.14 | (-0.13 – 0.42) | 0.47 | (0.21 – 0.73) | 0.045 |
|  |  | 2000 | 13.99 | (13.94 – 14.03) | -0.01 | (-0.24 – 0.22) | 0.56 | (0.34 – 0.78) | 0.038 |
| R_2,4_(θ) | GRASE | 1073 | 14.19 | (14.17 – 14.20) | 0.59 | (0.56 – 0.63) | - | - | 0.026 |
|  | CPMG | 1073 | 14.20 | (14.18 – 14.22) | 0.51 | (0.48 – 0.54) | - | - | 0.022 |
|  |  | 1500 | 13.91 | (13.88 – 13.94) | 0.60 | (0.54 – 0.66) | - | - | 0.045 |
|  |  | 2000 | 13.99 | (13.96 – 14.01) | 0.55 | (0.50 – 0.60) | - | - | 0.037 |
| R_2,5_(θ) | GRASE | 1073 | 14.10 | (14.05 – 14.15) | 0.61 | (0.54 – 0.69) | - | - | 0.053 |
|  | CPMG | 1073 | 14.13 | (14.08 – 14.17) | 0.53 | (0.46 – 0.60) | - | - | 0.049 |
|  |  | 1500 | 13.82 | (13.77 – 13.88) | 0.63 | (0.54 – 0.71) | - | - | 0.062 |
|  |  | 2000 | 13.91 | (13.85 – 13.97) | 0.57 | (0.47 – 0.66) | - | - | 0.064 |
